# Reliability and Effectiveness of a Lateral Countermovement Jump for Stratifying Shuffling Performance Amongst Elite Basketball Players

**DOI:** 10.3390/sports10110186

**Published:** 2022-11-21

**Authors:** Eric Leidersdorf, Jacob Rauch, Trent Reeves, Leah Borkan, Javan Francis, Luke Storey, Eduardo Oliveira De Souza, Marcus Elliott, Carlos Ugrinowitsch

**Affiliations:** 1 Peak Performance Project, Santa Barbara, CA 93101, USA; 2 University of California, Santa Barbara, Santa Barbara, CA 93106, USA; 3 Human Performance Laboratory, Health Sciences and Human Performance Department, University of Tampa Florida, Tampa, FL 33606, USA; 4 Laboratory of Adaptations to Strength Training, School of Physical Education and Sport University of São Paulo, São Paulo 05508-060, Brazil

**Keywords:** force plate, acceleration, shuffle, NBA, biomechanics, sport, change of direction

## Abstract

Though research suggests that basketball players spend approximately 31% of game actions shuffling laterally, limited data are available on the kinetic factors that separate fast and slow shufflers. The purpose of this study was twofold: (1.) Examine the reliability of kinetic metrics from a single-leg Lateral Countermovement Jump (LCMJ) (2.) Determine if kinetic metrics from the LCMJ can stratify above (i.e., “fast”) or below (i.e., “slow”) median shuffling performance. Twenty professional basketball players participated in the reliability study (21.7 ± 3.5 years, 1.98 ± 0.1 m; 89.9 ± 10.9 kg). One hundred seven professional and thirty-three collegiate basketball players (N = 140) (22.7 ± 3.5 years, 2.0 ± 0.1 m; 98.4 ± 11.9 kg) participated in the experimental study examining the ability of LCMJ kinetics to stratify shuffling performance. Reliability was assessed using Bland–Altman plots, coefficients of variation (CVs), typical errors (TEs), and intraclass correlation coefficients (ICCs). Anthropometric and LCMJ kinetic differences between fast and slow shufflers were assessed with an independent *t*-test. Four kinetic metrics (peak vertical force, peak lateral force, relative lateral force, and lateral impulse) met within- and between-session reliability thresholds (CV < 10% and ICC > 0.70). Faster shufflers generated significantly more relative lateral force than their slower counterparts (9.51 ± 0.8 Nx/kg vs. 8.9 ± 0.9 Nx/kg, %Diff 6.3, *p* < 0.00007, ES = 0.70). Basketball practitioners who have access to triaxial force plates may consider adding the LCMJ into their testing battery, as relative lateral force is a reliable metric that can stratify fast and slow shufflers.

## 1. Introduction

Basketball is a high-intensity intermittent sport that requires a variety of motor, cognitive, technical, and tactical demands [1]. During the technical execution of tactical actions, athletes are required to accelerate, decelerate, change direction, jump, and shuffle across varying distances and intensities [2,3]. On offense, acceleration, deceleration, and lateral change of direction (COD) ability may enable a player to create more space while driving with the ball or cutting without the ball. While on defense, the proper execution of these tasks may enable an athlete to close space more effectively, decreasing the likelihood of an open shot attempt. As the execution of these motor demands can play a pivotal role in winning or losing, sport scientists are constantly searching for the most effective methods to assess and develop them.

When compiling an assessment battery for elite basketball athletes, practitioners are faced with constraints such as space and time allotted to assess each athlete. Furthermore, competition demands may limit the amount of mechanical loading a practitioner is comfortable exposing an athlete to. Thereby, in addition to yielding reliable metrics that can stratify performance in key motor demands required during competition, assessments in elite basketball should be time efficient, require minimal familiarization, and limit mechanical loading. 

One of the most common practical assessments in elite basketball is the bilateral countermovement jump (CMJ) [4,5,6]. In addition to being a reliable method of measuring an athlete’s lower body power, recent findings have demonstrated that when performed on force platforms, kinetic metrics from the CMJ can differentiate high and low horizontal acceleration and deceleration performers amongst team sport athletes (e.g., soccer, rugby league, rugby union) using a median split analysis [7]. Though horizontal acceleration and deceleration are key motor demands in basketball, players are also required to accelerate and decelerate laterally [3,8]. In fact, research has demonstrated that basketball players spend up to 31% of their live game actions shuffling laterally [3]. Furthermore, preliminary research suggests that athletes who perform better in lateral cutting maneuvers display greater relative lateral force outputs when changing direction [8]. Despite this, no study has identified kinetic metrics able to stratify fast and slow shufflers. Thus, a practical laboratory test that could identify kinetic factors capable of stratifying lateral shuffling performance amongst elite basketball players is warranted.

Recently, kinetic variables from a single-leg lateral countermovement jump (LCMJ) demonstrated acceptable reliability amongst team sport athletes (e.g., soccer, basketball, field hockey, and rugby) and amateur hockey players [9,10]. However, there is currently no data available on the reliability or applications of an LCMJ in elite basketball athletes. Therefore, the purpose of this study was twofold: (1) To examine the within- and between-session reliability of kinetic metrics from an LCMJ amongst an elite basketball population; and (2) examine if kinetic metrics from the LCMJ can stratify fast and slow lateral shuffling performers. 

## 2. Materials and Methods

### 2.1. Experimental Approach to the Problem

For purpose number one, a within-subject experimental design was used to examine the within- and between-session reliability of kinetic metrics from the LCMJ (Table 1 and Table 2). Participants reported to the laboratory at the same time of day, for three separate sessions separated by 48–72 h. Testing consisted of one submaximal and two maximal LCMJs per leg during each session. Session one served as a familiarization session whereas sessions two and three were used to examine the within- and between-session reliability. 

**Figure 1:**
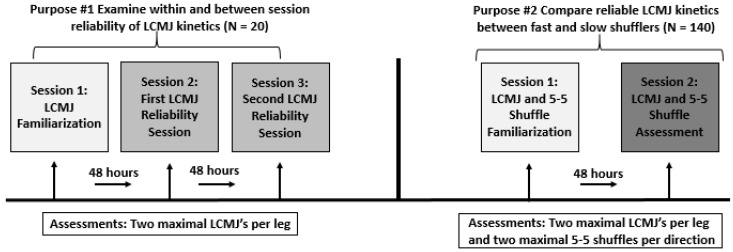
Experimental Design.

For purpose number two, a cross-sectional experimental design was used to compare reliable LCMJ kinetics between fast- and slow-shuffling performers. Thirty days after purpose one was completed, a separate cohort of participants reported to the laboratory for two sessions, at the same time of day, interspersed by at least 48 h. Participants performed one submaximal and two maximal LCMJs per leg along with one submaximal and two maximal 5-5 shuffles per leg. Session one served as a familiarization session, while data from session two was extracted for further analysis (Figure 1). 

### 2.2. Participants

Twenty professional basketball players volunteered to participate in the reliability portion of the study (21.7 ± 3.5 years, 1.98 ± 0.1 m; 89.9 ± 10.9 kg). One hundred seven professional (National Basketball Association, G-League, EuroLeague) and thirty-three collegiate basketball players (NCAA Division 1) (N = 140) (22.7 ± 3.5 years, 2.0 ± 0.1 m; 98.4 ± 11.9 kg) volunteered to participate in the experimental portion of the study. To be eligible for inclusion, all participants had to be on an active professional or division one collegiate roster and free from any musculoskeletal injuries at the time of data collection. If participants performed a strength training or on-court workout 48 h prior to the testing session, they were excluded from the analysis. All testing procedures took place during the participants’ offseason (April–September). All participants signed an informed consent form going over the details of the procedures and inherent risks. The study was approved by the University of Tampa Institutional Review Board (ID: 20006548).

### 2.3. Testing Procedures

Participants arrived at the laboratory at 9:00 AM for each testing session. After being briefed on the procedures, participants underwent a standardized warm-up which consisted of a series of 16 dynamic movements performed on a twenty-yard indoor track. Movements included two lunge and hinge variations and progressed to four low-intensity plyometric exercises (ankle skips, drop skips, cross-over skips, and A-skips). After the standardized warm-up, participants were brought over to the testing area which consisted of a nine-camera Simi Motion (Simi Reality Motion Systems GmbH, Unterschleissheim, Germany) analysis system in conjunction with two triaxial Bertec (Model No. 6090, Bertec Corporation, Columbus, OH, USA) force platforms. The motion capture system synchronized motion (120 Hz) and force data (1000 Hz). Final data points were analyzed and exported using a custom-built code in MATLAB (The MathWorks, Inc., Natick, MA, USA). The participants’ weight, height, and dominant jumping leg were recorded by the principal investigator. For the reliability study, participants then performed the LCMJ. For the experimental study, participants then performed the LCMJ and 5-5 shuffle following a six-minute rest period.

### 2.4. Assessments

#### 2.4.1. Lateral Countermovement Jump (LCMJ)

**Figure 2:**
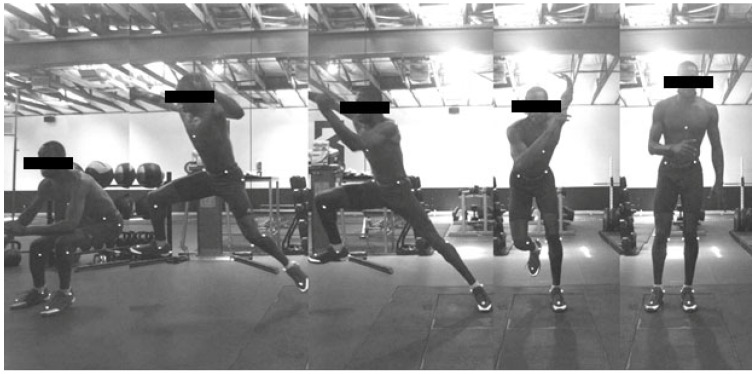
Example LCMJ.

For the LCMJ, participants were asked to start with both feet on a single force platform and stand still for a three-second weighing phase. Afterward, they were instructed to push as hard as they could off a single leg, jumping lateral to medial, away from the platform (Figure 2). Participants could utilize whatever movement strategy they preferred; however, in an effort to reduce excessive movement in the transverse plane, participants were instructed to not cross their lead leg behind the drive leg. Participants performed one submaximal jump followed by two maximal jumps per leg. Data were discarded if the outside lead leg crossed behind the drive leg as evidenced from the video recording of each rep. 

#### 2.4.2. 5-5 Shuffle

**Figure 3:**
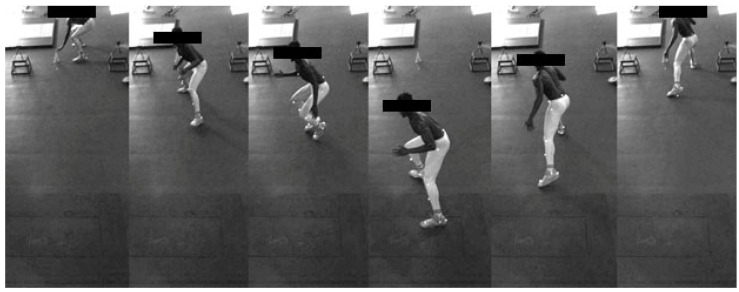
Example 5-5 Shuffle.

Data collection procedures for the 5-5 shuffle were adapted from Shimokochi et al., (2013) [8]. Participants started in a three-point stance 30 cm behind a pair of Brower timing gates set at a height of 40 cm (Brower Timing Systems, Draper, UT, USA). On their own volition, participants were instructed to perform a lateral shuffling maneuver as they would perform during a defensive play in basketball. Participants were instructed to shuffle for 5 m to a clearly identifiable marking on the floor, whereby they would make a lateral COD and shuffle back through the starting line in as short amount of time as possible (Figure 3). Each participant performed a submaximal warm-up trial followed by two maximal attempts in each direction. Performance for the 5-5 shuffle was determined by the time (s) it took the participant to shuffle back through to the starting position for a total distance traveled of 10 m. Data were discarded if the participant crossed their feet during the shuffle or did not shuffle for the full distance as evidenced from the video recording of each rep. Within-session reliability for the 5-5 shuffle was determined on a sub-sample of the recruited players prior to the experimental study. The shuffling task met predetermined reliability thresholds for both directions (N = 20, dominant side 0.9% coefficient of variation (CV), 0.03 typical error (TE), 0.9 intraclass correlation coefficient (ICC); non-dominant side 1.4% CV, 0.05 TE, 0.83 ICC).

### 2.5. Data Analysis

For the LCMJ, the following variables were collected for further analysis: peak vertical and lateral force, relative peak vertical and lateral force, vertical rate of force development, lateral rate of force development, relative net force, lateral impulse, relative lateral impulse, and total movement time. The initiation of the movement was defined as a drop in vertical force of 2 standard deviations (SD) below average body weight. The relative lateral force (Nx/kg) was determined by taking the maximum force (N) achieved in the X (lateral) axis during the movement and dividing this value by the participant’s body weight in kilograms. The relative vertical force (Nz/kg) was determined by normalizing the maximum force (N) in the Z (vertical) axis during the movement and dividing this value by the participant’s body weight in kilograms [8]. The average vertical and lateral rates of force development (N·s^−1^) were determined by taking the peak forces in each respective axis (Nz, Nx) and dividing these values by the time to peak force (s). The net force was determined by the resultant force vector of force generated in the vertical and lateral directions. Net relative lateral impulse (Ns/kg^−1^) was determined by taking the integral of the force–time curve divided by the participant’s body weight in kilograms. Total movement time (s) was determined by subtracting the time at take-off (Nz < 10 N) from the time at the initiation of the movement [6]. For within-session reliability, both repetitions from each leg were analyzed from session two of the reliability study. For between-session reliability, the single rep from each leg with the greatest lateral force (Nx) between sessions two and three of the reliability study was used for further analysis. For the experimental study, the single reps with the greatest lateral force (Nx) for the dominant and non-dominant leg from session two of the experimental study were averaged and used for further analysis. For the 5-5 shuffle, the best repetition (i.e., fastest time to completion (s)) from each leg was averaged and used for further analysis from session two of the experimental study.

### 2.6. Statistical Analysis

Within- and between-session reliability was assessed using Bland–Altman plots to represent the mean difference, along with the upper and lower limits of agreement (LOA), between trials and sessions. Additionally, the coefficient of variation (CV), typical error (TE), and intraclass correlation coefficients (ICCs) were also reported on. The CV was calculated by dividing the population standard deviation by the population mean, averaged across each trial. The TE was calculated by dividing the standard deviation of the difference between trial 1 and 2 and dividing it by the square root of two. Reliability thresholds for the CV and ICC were set in accordance with previous literature conducted on similar populations (CV < 10% and ICC > 0.70) [9,11]. As reliability was similar for the dominant and non-dominant leg, only the dominant leg findings are reported [9]. COD performance was calculated by averaging the best 5-5 shuffle time from each direction. Participants were then dichotomized as “fast” or “slow” based on a median split of shuffling performance [7,12]. After normality (i.e., Shapiro–Wilks) and variance assurance (i.e., Levene), an independent *t*-test with a Bonferroni correction was run to examine anthropometric and reliable LCMJ metric differences between fast and slow COD performers [13]. As seven variables were tested, the significance level was set at *p* < 0.0071, and data were presented as mean, SD, percent difference, and Cohen’s d effect size utilizing the pooled SD. Effect sizes of 0.2 ≤ d < 0.5 were interpreted as small, 0.5 ≤ d < 0.8 as medium, and d ≥ 0.8 as large [14]. Data were analyzed using RStudio 2021.09.1 (RStudio Team (2021). RStudio: Integrated Development for R. RStudio Boston, MA USA).

## 3. Results

### 3.1. Reliability 

**Figure 4:**
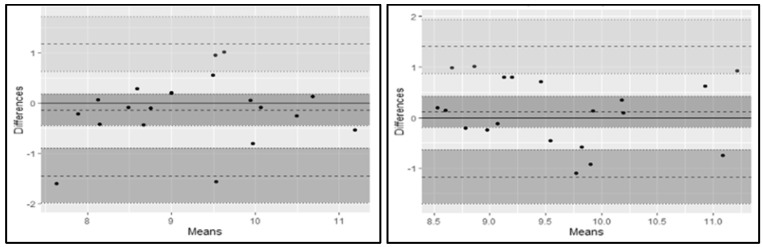
Legend. Within (**left**)- and Between (**right**)-session Bland–Altman plots of Relative Lateral Force on the Dominant Leg.

The complete results for the within-session reliability data can be found in Table 1. In brief, nine of the ten metrics demonstrated sufficient within-session reliability (i.e., CV < 10% and ICC > 0.70). Vertical rate of force development (CV = 10.2%) was the only metric that did not meet the predetermined reliability threshold. The complete results for the between-session reliability data can be found in Table 2. In brief, only four out of ten metrics demonstrated adequate between-session reliability (peak vertical force, peak lateral force, relative lateral force, and lateral impulse). The within and between session Bland–Altman plots of relative lateral force on the dominant leg can be found in Figure 4.

### 3.2. Independent t-test Comparing Average Shuffling Performance between “Fast” and “Slow” Participants

Participants categorized as fast demonstrated significantly lower shuffle times (2.67 ± 0.07 s vs. 2.87 ± 0.08 s, %Difference (Diff) −7.22, *p* < 0.0000, ES = 2.67) compared to the participants categorized as slow (Table 3).

### 3.3. Independent t-test Comparing Anthropometrics and LCMJ Kinetics between “Fast” and “Slow” Participants 

The complete results for the independent *t*-test comparing anthropometric measurements and LCMJ differences between participants with fast and slow lateral shuffle times can be found in Table 3. In brief, relative lateral force (9.51 ± 0.80 Nx/kg vs. 8.93 ± 0.87 Nx/kg, %Diff 6.29, *p* = 0.00007, ES = 0.70) was the only variable that was significantly different between fast and slow participants.

## 4. Discussion

The first aim of our study was to investigate the within- and between-session reliability of kinetic metrics derived from an LCMJ amongst a cohort of elite basketball athletes. Our findings demonstrated that nine of the ten investigated variables were considered acceptable for within-session reliability (CV < 10% and ICC > 0.70) [9,11]. However, only four variables demonstrated acceptable between-session reliability. A secondary aim was to examine if kinetic metrics derived from the LCMJ could differentiate fast and slow performers in a lateral shuffling task. Here, our findings revealed that faster lateral shufflers produced significantly greater amounts of relative lateral force in the LCMJ compared to their slower counterparts.

Regarding the within-session reliability of peak vertical force and peak lateral force, our findings (CVs = 2.72%,3.72%) agreed with those of Meylan et al., (2010) who reported average within-session CVs (3.8%,7.35%) of 30 team sport athletes (e.g., soccer, basketball, field hockey, and rugby) performing three LCMJs across two time points [9]. Our between-session reliability data for peak vertical force and peak lateral force (ICC = 0.78, 0.85) were also in agreement with the values reported by Meylan et al., (2010) (ICC= 0.96,0.89) [9], and Donskov et al., (2021) who examined the between-session reliability of twelve kinetic metrics derived from the LCMJ amongst a cohort of amateur male hockey players (e.g., peak vertical force ICC Left leg (L) = 0.98; Right leg (R) = 0.97, peak lateral force ICC L = 0.91: R = 0.98) [10]. It appears that peak vertical force and peak lateral force display acceptable within- and between-session reliability across varying sporting populations (i.e., hockey and other team sport athletes). However, our data suggest that temporal variables such as vertical and lateral rates of force development may not be reliable between sessions. Rates of force development have historically demonstrated lower test-to-test reliability results compared to peak force outputs across other practical laboratory tests (i.e., counter movement jump) [15,16]. This suggests that athletes may be able to reach peak forces with a high degree of consistency between trials and sessions, but the temporal measures associated with these movements between trials and sessions should be interpreted with caution. As such, it may behoove practitioners to report solely on the aforementioned reliable metrics if they are considering adding an LCMJ to their testing battery until more data become available.

A secondary aim of our study was to investigate if kinetic metrics derived from the LCMJ could stratify fast and slow lateral shuffling performers. Our findings demonstrated that faster performers in the lateral shuffling task tended to produce more relative lateral force in the LCMJ compared to their slower counterparts. Historically, it has been difficult to compare the findings in the literature examining relationships between practical laboratory tests and COD performance, as numerous COD tests have been administered across varying sporting populations with different approach velocities, numbers of direction changes, and angles of direction change [17]. Furthermore, common COD assessments such as the T-Agility Test may not offer basketball practitioners relevant insights as the sprinting and shuffling distances exceed those during basketball game play [12,18]. As such, researchers have shortened the distance of the T-Agility Test (i.e., Modified T-Agility Test (MAT)) to better reflect the multidirectional, high-intensity movements experienced during COD maneuvers in team sports like basketball [12,18]. The MAT consists of a 5 m linear sprint to a cone, followed by shuffling 2.5 m to the left or the right to the next cone, followed then by a 5 m shuffle to a cone in the opposite direction, ending with a 2.5 m shuffle back to center and a 5 m backpedal through the starting gate. As the longest distance (10 m) is covered by a shuffle, it has been suggested that the MAT time can be mostly related to lateral movement efficiency [18]. Scanlan et al., (2019) examined relationships between laboratory power assessments and MAT performance amongst national and state-level adolescent male basketball players (N = 2 4, 17.3 ± 0.5 years, 1.92 ± 0.11 m, 84.0 ± 10.6 kg). These researchers performed a median split analysis similar to the one utilized in our investigation and reported that athletes with faster MAT times performed better in a standing long jump and produced greater relative peak force during an isometric mid-thigh pull and CMJ [12]. Interestingly, these researchers did not find any relationships between repeated lateral bound distance and MAT performance. These researchers note that this may be due to the low variance in lateral bound distance amongst the sample (Full Sample 9.32 ± 0.72 m, Fast 9.37 ± 0.79 m, Slow 9.26 ± 0.68 m). Thus, lateral bound distance (1.18% difference between fast and slow athletes) in this population may not be sensitive enough to detect differences in MAT performance. This provides further support for measuring the underlying kinetics of a lateral bound, as they may be more sensitive to differences compared to distance alone. For example, the fast and slow shufflers in our investigation demonstrated a 6.29% difference in relative lateral force. Nevertheless, comparisons between our investigations should be taken with caution, as we had different levels of athletes (i.e., collegiate and professional vs. adolescent) and assessments (5-5 shuffle vs. MAT, LCMJ vs. repeated lateral bound). Furthermore, though the distances in the MAT are more reflective of the COD distances required in basketball compared to the traditional *t*-Test (as it requires linear sprinting and backpedaling), it may not be the best assessment to isolate lateral COD ability as compared to the 5-5 shuffle utilized in our investigation. 

To the authors’ knowledge, Shimokochi et al., (2013) is the only other study to implement an isolated shuffling assessment. This group examined the kinetic and kinematic factors related to lateral quickness. These researchers reported that collegiate female basketball players (N = 28, 20.7 ± 1.0 years, 166.0 ± 8.5 cm, 58.7 ± 7.5 kg) who exhibited greater lateral force and a lower center of mass performed better in a lateral cutting maneuver [8]. Collectively, our findings suggest that relative lateral force may be an important quality for lateral shuffling performance.

While it is generally accepted that successful COD performance requires a variety of physical (eccentric, concentric, reactive strength) and technical factors (coordination, trunk positioning, angle of force distribution) [19,20,21,22], the reliance on each factor will likely change depending upon the specific COD demands [23]. Though the LCMJ is not all-inclusive, it may be a way for basketball practitioners to objectively measure some of the physical parameters required for lateral shuffling performance. Furthermore, the LCMJ may have a practical upside as it is time efficient, requires minimal familiarization, and requires negligible amounts of mechanical loading compared to traditional COD batteries. These practical considerations may maximize effort from the athlete and increase the frequency with which an athlete is willing to test, providing practitioners with the ability to track changes over time. 

Our project had several inherent limitations that need to be addressed. Due to the nature of our study design (i.e., cross-sectional), it was difficult to account for every confounding factor that may have impacted the results. For instance, athletes came into the facility at various time points in their off-season and as a result we were not able to control for training history at the time of their assessment. Nevertheless, we tried to recruit a sufficient sample size (i.e., 140) to accurately represent the elite basketball population at large. Additionally, we tried to account for anthropometric differences by normalizing certain kinetic values based on body mass. Future studies should investigate relationships between kinetic metrics from the LCMJ and kinetic or kinematic metrics from the lateral 5-5 shuffle test, as these metrics may encompass additional factors integral to COD performance. Furthermore, future studies should also compare different training programs and their effectiveness for improving relative lateral force and shuffling performance. Lastly, our findings are only generalizable to the specific 5-5 shuffle assessment utilized in our study design. Future studies should investigate the relationships between on-court lateral performance and performance in the LCMJ and in the 5-5 shuffle.

## 5. Conclusions

Basketball practitioners who have access to triaxial force plates may consider adding the LCMJ to their testing battery, as relative lateral force is a reliable metric that can stratify fast and slow shufflers. Furthermore, the LCMJ may have additional practical implications as it is time efficient, requires minimal familiarization, and incentivizes maximum output while limiting mechanical loading. Though the LCMJ is not all-inclusive, it can serve as a supplement to more traditional laboratory tests (i.e., CMJ) and provide practitioners with insights regarding an athlete’s lateral force production capabilities. Practitioners can then develop their own population norms with kinetic metrics from the CMJ and LCMJ, and use objective data to decide if an athlete needs to work on vertical and/or lateral force production characteristics. Similarly, if an athlete exhibits sufficient relative lateral force production and poor shuffling performance, this may indicate that they need to develop other physical (i.e., eccentric strength) or technical factors (i.e., trunk positioning and coordination). Lastly, for practitioners who do not have triaxial force plates, our findings may simply urge them to add in exercises that target lateral force production (i.e., assisted and resisted lateral bounds, lateral sled drags, and lateral lunges), as our data suggest relative lateral force is an important quality for performing lateral shuffling maneuvers.

## Figures and Tables

**Table 1:** Within-session reliability for dominant leg LCMJ metrics (Mean + SD).

Variables	Trial 1	Trial 2	Bias	Upper LOA	Lower LOA	CV	TE	ICC
Peak Vert F (Nz)	1746.57 ± 198.21	1713.34± 1 95.57	33.24	198.74	−132.26	2.72	59.71	0.90
Rel Peak Vert F (Nz/kg)	9.42 ± 1.90	9.23 ± 1.80	0.19	2.34	−1.96	6.90	0.78	0.83
Peak Lat F (Nx)	829.24 ± 120.09	841.32 ± 110.96	−12.09	111.71	−135.88	3.72	44.66	0.85
Rel Peak Lat F (Nx/Kg)	9.17 ± 1.08	9.31 ± 0.98	−0.13	1.18	−1.45	3.72	0.47	0.79
Rel Net F (Nz + Nx/kg)	13.23 ± 1.57	13.16 ± 1.62	0.07	2.09	−1.95	4.48	0.73	0.80
Vert RFD (Nz/s)	3746.91 ± 1393.65	3626.42 ± 1397.80	120.49	1536.62	−1295.65	10.2 *	510.90	0.87
Lat RFD (Nx/s)	1581.30 ± 404.77	1553.23 ± 386.94	28.08	618.77	−562.61	9.12	213.10	0.72
Lat IMP (N·s)	241.85 ± 39.14	248.88 ± 43.36	−7.04	19.64	−33.72	3.47	9.63	0.88
RelLatImp (N·s/kg^−1^)	2.67 ± 0.32	2.74 ± 0.37	−0.08	0.23	−0.38	3.47	0.11	0.88
TMT (s)	0.66 ± 0.15	0.68 ± 0.14	−0.02	0.19	−0.23	7.29	0.08	0.72

Legend. First Row: Trial 1, Trial 2, Bias (Bland–Altman), Upper Limit of Agreement (Bland–Altman, Lower Limit of Agreement (Bland–Altman, Coefficient of Variation, Typical Error, Intraclass Correlation Coefficient. First Column: Peak Vertical Force, Relative Vertical Force, Peak Lateral Force, Relative Lateral Force, Relative Net Force, Vertical Rate of Force Development, Lateral Rate of Force Development, Lateral Impulse, Relative Lateral Impulse, Total Movement Time. (*) Indicates values below a predetermined reliability threshold (CV< 10% and ICC >0.70).

**Table 2:** Between-session reliability for dominant leg LCMJ metrics (Mean + SD).

Variables	Trial 1	Trial 2	Bias	Upper LOA	Lower LOA	CV	TE	ICC
Peak Vert F (Nz)	1750.92 ± 192.54	1775.00 ± 236.67	−24.08	260.18	−308.35	4.79	102.55	0.78
Rel Vert F (Nz/kg)	9.57 ± 2.13	9.57 ± 1.77	0.00	3.07	−3.06	10.05 *	1.10	0.70
Peak Lat F (Nx)	876.34 ± 107.77	872.12 ± 113.08	4.22	127.25	−118.80	4.46	44.38	0.85
Rel Lat F (Nx/Kg)	9.62 ± 0.86	9.58 ± 0.98	0.13	1.49	−1.23	4.46	0.49	0.72
Rel Net F (Nz + Nx/kg)	13.68 ± 1.73	13.58 ± 1.40	0.10	2.72	−2.51	5.44	0.94	0.66 *
Vert RFD (Nz/s)	3296.12 ± 924.91	3611.39 ± 1376.28	−315.27	3062.57	−3693.11	27.01 *	1218.62	0.08 *
Lat RFD (Nx/s)	1496.43 ± 331.59	1575.35 ± 400.40	−78.93	1110.32	−1268.18	22.09 *	429.04	−0.34 *
Lat IMP (N·s)	256.70 ± 47.18	254.42 ± 40.92	2.28	60.33	−55.76	6.92	20.94	0.79
RelLatImp (N·s/kg^−1^)	2.81 ± 0.29	2.78 ± 0.38	0.03	0.66	−0.59	6.78	0.23	0.58 *
TMT (s)	0.72 ± 0.17	0.69 ± 0.14	0.04	0.53	−0.46	19.64 *	0.18	−0.24 *

Legend. First Row: Trial 1, Trial 2, Bias, Upper Limit of Agreement, Lower Limit of Agreement, Coefficient of Variation, Typical Error, Intraclass Correlation Coefficient. First Column: Peak Vertical Force, Relative Vertical Force, Peak Lateral Force, Relative Lateral Force, Relative Net Force, Vertical Rate of Force Development, Lateral Rate of Force Development, Lateral Impulse, Relative Lateral Impulse, Total Movement Time. (*) Indicates values below a predetermined reliability threshold (CV < 10% and ICC > 0.70).

**Table 3:** Anthropometric and LCMJ differences between participants with Fast and Slow Lateral Shuffle Times (Mean + SD).

Variables	Fast N = 70	Slow N = 70	% Diff	ES	*p*-Value
AVG 5-5 Shuffle (s)	2.67 ± 0.07	2.87 ± 0.08	7.22%	2.67	0.0000 *
BM (kg)	96.07 ± 11.03	100.72 ± 12.39	4.73%	0.39	0.0217
Height (cm)	198.84 ± 7.85	201.71 ± 7.89	1.43%	0.37	0.0325
Peak Vert N (Nz)	1792.2 ± 249.1	1807.3 ± 255.2	0.83%	0.06	0.7240
Peak Lat N (Nx)	909.4 ± 107.7	903.1 ± 112.1	0.65%	0.06	0.7363
Rel Lat N (Nx/kg)	9.51 ± 0.8	8.93 ± 0.87	6.29%	0.70	0.0000 *
Lat Imp (N·s)	252.20 ± 39.21	257.76 ± 38.16	2.18%	0.24	0.3979

First Column: Average 5-5 Shuffle, Body Mass, Height, Peak Vertical Force, Peak Lateral Force, Relative Lateral Force, Lateral Impulse. Bonferroni correction-established *p*-value threshold of *p* < 0.0071 *.
